# Astaxanthin inhibits apoptosis in alveolar epithelial cells type II *in vivo and in vitro* through the ROS-dependent mitochondrial signalling pathway

**DOI:** 10.1111/jcmm.12347

**Published:** 2014-09-12

**Authors:** Xiaodong Song, Bingsi Wang, Shengcui Lin, Lili Jing, Cuiping Mao, Pan Xu, Changjun Lv, Wen Liu, Ji Zuo

**Affiliations:** a Department of Cellular and Genetic Medicine, School of Basic Medical Sciences, Fudan UniversityShanghai, China; b Medicine Research Center, Binzhou Medical UniversityYantai, China; c Department of Respiratory Medicine, Affiliated Hospital to Binzhou Medical UniversityYantai, China; d Department of Pathology, Affiliated Hospital to Binzhou Medical UniversityYantai, China; e Department of Respiratory Medicine, Affiliated Hospital to Binzhou Medical UniversityBinzhou, China

**Keywords:** lung fibrosis, oxidative stress, astaxanthin, ROS, mitochondrial signalling pathway

## Abstract

Oxidative stress is an important molecular mechanism underlying lung fibrosis. The mitochondrion is a major organelle for oxidative stress in cells. Therefore, blocking the mitochondrial signalling pathway may be the best therapeutic manoeuver to ameliorate lung fibrosis. Astaxanthin (AST) is an excellent antioxidant, but no study has addressed the pathway of AST against pulmonary oxidative stress and free radicals by the mitochondrion-mediated signalling pathway. In this study, we investigated the antioxidative effects of AST against H_2_O_2_- or bleomycin (BLM)-induced mitochondrial dysfunction and reactive oxygen species (ROS) production in alveolar epithelial cells type II (AECs-II) *in vivo and in vitro*. Our data show that AST blocks H_2_O_2_- or BLM-induced ROS generation and dose-dependent apoptosis in AECs-II, as characterized by changes in cell and mitochondria morphology, translocation of apoptotic proteins, inhibition of cytochrome c (Cyt c) release, and the activation of caspase-9, caspase-3, Nrf-2 and other cytoprotective genes. These data suggest that AST inhibits apoptosis in AECs-II cells through the ROS-dependent mitochondrial signalling pathway and may be of potential therapeutic value in lung fibrosis treatment.

## Introduction

Lung fibrosis is associated with inflammation characterized by the recruitment of macrophages, neutrophils and lymphocytes in the airways 1. However, the cellular and molecular mechanisms underlying the pathogenesis of lung fibrosis are largely unclear. To date, no effective therapy can prevent or reverse pulmonary fibrogenesis, which indicates the need to identify new molecular targets and drugs 2. Oxidative stress is an important molecular mechanism underlying lung fibrosis. Accumulated evidence suggests that reactive oxygen species (ROS), such as superoxide, hydrogen peroxide (H_2_O_2_), peroxynitrite and hydroxyl radical, are major mediators of lung inflammatory processes. Activated phagocytes release large amounts of ROS that induce tissue injury and inhibit tissue repair, leading to lung fibrosis 3–5. However, the causal role of ROS released from environmental exposures and inflammatory/interstitial cells in mediating fibrosis, as well as the most efficient mechanism to target an imbalanced ROS production in patients with fibrosis, has not been firmly established. The mitochondrion, a major organelle, has attracted much interest because of its involvement in ROS production and oxygen consumption in cells 6. Thus, blocking the mitochondrial signalling pathway may be the best therapeutic manoeuver to ameliorate fibrosis 7.

Astaxanthin (AST) is the only known ketocarotenoid that can be transported into the brain by transcytosis through the blood-brain barrier 8. In addition to its excellent antioxidant activity, AST possesses anti-inflammatory and anti-tumour effects 9–12. AST has an elimination half-life of 15.9 ± 5.3 hrs (*n* = 32) 13. Several studies 14,15 have shown that AST provides important metabolic functions such as conversion into vitamin A, enhancement of the immune response, and protection against diseases such as cancer by scavenging oxygen radicals. Lee *et al*. 16 demonstrated that AST protects against MPTP/MPP+-induced neuronal mitochondrial damage by ROS *in vivo and in vitro* as well as inhibits ROS generation and mitochondrial membrane potential (MMP) collapse induced by MPP+ in SH-SY5Y cells. The cytoprotection of AST against MPTP/MPP+-induced cell death may be associated with the attenuation of oxidative damage by inhibiting ROS generation and with the prevention of MMP collapse. These results suggest that the balance between generating and scavenging of free radicals is important for cell or animal survival. However, to our knowledge, no study has addressed the protection of AST against pulmonary oxidative stress and free radicals *via* a mitochondrion-mediated signalling pathway.

We have studied the anti-fibrotic effect of AST and reported that AST could relieve the symptoms and stop the progression of lung fibrosis in the later lung fibrosis 17, but we could not investigate its antioxidant effect in the early lung fibrosis. In this study, we investigated the mitochondrion-mediated antioxidative effect of AST in alveolar epithelial cells type II (AECs-II) in the early lung fibrosis. The data showed that AST could inhibit AECs-II apoptosis *in vivo and in vitro* through the ROS-dependent mitochondrial signalling pathway.

## Materials and methods

### Human tissue samples

Lung tissue samples were obtained by open lung biopsy. Pulmonary fibrosis was diagnosed according to the American Thoracic Society/European Respiratory Society consensus criteria 18, including clinical, radiographic and characteristic histopathological features (*n* = 5). Control non-pulmonary fibrosis lung tissue samples were obtained from smokers who underwent thoracic surgery for localized primary lung carcinoma (*n* = 5). The local ethics committee approved the study, and patients gave their informed consent before lung surgery.

### Cell lines and reagents

AST was dissolved in dimethyl sulfoxide (Sigma-Aldrich, St Louis, MO, USA) to yield a 10 mM stock solution as our previously described 11,12. Rat lung epithelial-T-antigen negative (RLE-6TN) cell line was purchased from the Cell Bank of the Chinese Academy of Sciences (Shanghai, China). The cell line was derived from AECs-II isolated from a 56 day old male F344 rat using airway perfusion with a pronase solution, which exhibits characteristics of AECs-II such as lipid-containing inclusion bodies and expression of cytokeratin 8 and 19. Pharmaceutical grade BLM was purchased from Nippon Kayaku Co., Ltd. (Tokyo, Japan). MMP detection kit was purchased from Beyotime Institute of Biotechnology (Haimen, China). Antibodies against Bcl-2, Bcl-XL, Bax, Bad, cytochrome c (Cyt c), Puma, Nuclear factor erythroid 2-related factor 2 (Nrf-2), P53 were purchased from Santa Cruz Biotechnology, Inc (Santa Cruz, CA, USA).

### Animal model

Sprague–Dawley (SD) rats with a mean weight of 200 g were provided by the Green Leaf Experimental Animal Center (Yantai, China). All animal experiments were performed in accordance with regulations established by the Committee on the Ethics of Animal Experiments of Binzhou Medical University. The rats were housed under a 12 hrs light/dark cycle, and allowed free access to food and water. 40 SD rats were randomly divided into four groups (10 rats each) including the sham group as our previously described 17, BLM-induced group (BLM group), AST treatment I group (1 mg/ml, 1 ml/kg) and AST treatment II group (2 mg/ml, 1 ml/kg). Lung fibrosis was induced by a single intratracheal instillation of 5 mg/kg BLM in 0.3 ml of saline in all groups except the sham group. The sham group received an equal volume of saline. After BLM treatment, rats in AST I and II groups received oral AST once daily. On day 7 all rats were killed and lung tissue sections were collected and immediately frozen in liquid nitrogen for further studies as our previously described 19.

### Sulforhodamine B (SRB) assay

Sulforhodamine B binds to basic amino acids of cellular proteins and colorimetric evaluation provides an estimate of total protein mass which is related to cell number. 1 × 10^5^ cells/ml at the logarithmic growth phase were digested with 0.25% trypsin and seeded into 96-well culture plates. After overnight incubation, the cells were incubated with medium containing different concentrations of H_2_O_2_ or AST. Then 50 μl of trichloroacetic acid was added to each well for additional 1 hr at 4°C. The plate was washed five times with double distilled water and dried under room temperature. The trichloroacetic acid-fixed cells were stained with 100 μl of 4 g/l SRB for 10 min., then the plate was washed five times with 1% acetate and air-dried overnight. The resulting crystals formed were dissolved with 150 μl of 10 mmol/l Tris (hydroxymethyl) aminomethane hydrochloride (Tris-HCl). Absorbance was measured with a microplate reader (SpectraMax M2, Sunnyvale, CA, USA) at 560 nm reference wavelength.

### Haematoxylin and eosin staining

Haematoxylin and eosin staining agents were purchased from Sigma-Aldrich. Lung tissues were fixed by instilling 4% paraformaldehyde through the trachea and were embedded in paraffin. Transverse sections of 4 mm thickness were stained with haematoxylin and eosin following the manufacturer’s standard protocol.

### Transmission electron microscopy (TEM) observation

Lung tissues or cells were fixed by treatment with fresh 3% glutaraldehyde at 4°C for at least 4 hrs, post-fixed in 1% osmium tetroxide for 1.5 hrs, dehydrated in gradient ethanol, infiltrated with Epon812, embedded and cultured at 37, 45 and 60°C for 24 hrs. Ultrathin sections prepared with an ultracut E ultramicrotome were stained with uranyl acetate and lead citrate and observed using a JEM-1400 TEM system from Jeol Ltd. (Tokyo, Japan) as our previously described 20.

### TdT-mediated dUTP-biotin nick end labelling (TUNEL) assay

Lung tissue sections were fixed in 4% paraformaldehyde, rinsed with PBS solution, incubated with 0.5% TritonX-100 for 20 min. Endogenous peroxidase activity was blocked using 3% H_2_O_2_ in 10 mM Tris. The treated tissues were placed in equilibration buffer and incubated with TdT enzyme in a humid chamber at 37°C for 2 hrs. The reaction was terminated with stop buffer and incubated with a biotin-conjugated rat anti-digoxin antibody and SABC-FITC (1:100; Boster Bio-Engineering Limited Company, China) for 30 min. respectively. Hoechst 33258 fluorochrome (Sigma-Aldrich) was used for nuclear staining. After washing with PBS, tissue sections were mounted in neutral glycerine and analysed under a laser scanning confocal microscope (Leica, Wetzlar, Germany). The fluorescence imaging was conducted with excitation at 488 nm and emission at 510 nm at room temperature.

### Apoptosis assay by flow cytometry

Suspended and adherent treated cells (1 × 10^6^) were collected and washed with cold PBS. The fixation fluid was washed with PBS. 500 μl Binding Buffer was added to re-suspend cells. 5 μl Annexin V-FIFC and 5 μl propidium iodide staining solution were respectively added for 20 min. staining away from light. Apoptosis rate was measured by flow cytometry (Beckman, Fullerton, CA, USA).

### Mitochondrial membrane potential assay

Mitochondrial membrane potential was determined by the 5,5′,6,6′-tetrachloro-1,1′,3,3′-tetraethylbenzimi dazolycarbocyanine iodide (JC-1; Beyotime Biotechnology) staining following the manufacturer’s instructions. Briefly, the harvested RLE-6TN cells were re-suspended in the mixture of 500 μl culture medium and 500 μl JC-1 (5 μg/ml) staining fluid, and then incubated in the dark at 37°C for 20 min. After washing with ice-cold staining buffer twice by centrifugation, cells were re-suspended in 500 μl culture medium and analysed by flow cytometry. The values of MMP staining from each sample were expressed as red and green fluorescence intensity.

### Assay of ROS generation

The levels of intracellular ROS were estimated using the membrane-permeable fluorescent probe 2,7-dichlorofluorescin diacetate (Beyotime Biotechnology). The assay was performed as the manufacturer’s protocol.

### Superoxide dismutase (SOD) activity

The activity of antioxidant enzyme SOD in RLE-6TN cells or lung was determined by using a Total Superoxide Dismutase Assay Kit with WST-1 (Beyotime Biotechnology) according to the manufacturer’s protocol. Briefly, after cells or lung were washed, lysed and vortexed, the supernatant was extracted. Sample, WST and enzyme-working solutions were prepared and added into the 96-well plate. The mixtures were incubated at 37°C for 20 min., and the absorbance was finally determined at 450 nm using a microplate reader (SpectraMax M2) 21.

### Catalase activity

Catalase activity was determined by using a Catalase Assay Kit (Beyotime) according to the manufacturer’s protocol. Briefly, the cells were washed with PBS and lysated using cell lysis buffer (20 mM Tris pH 7.5, 150 mM NaCl, 1% Triton X-100, 2.5 mM sodium pyrophosphate, 1 mM EDTA, 1% Na3VO4, 0.5 mg/ml leupeptin, and 1 mM phenylmethane- sulfonyl fluoride).The lysates were centrifuged at 10,000 × g at 48°C for 5 min., and the supernatants were collected to determine enzyme activity. The assays were performed on the microplate reader (SpectraMax M2) at 520 nm for catalase. Protein concentrations were determined using BCA protein assay.

### Caspase activity

Caspase-3 and -9 activities were measured by cleavage of chromogenic caspase substrates, Ac-DEVD-pNA (acetyl-Asp–Glu–Val–Asp p-nitroanilide) and Ac-LEHD-pNA (acetyl-Leu–Glu–His–Asp p-nitroanilide) by an assay kit (Beyotime Biotechnology). Briefly, cell lysate from 1 × 10^6^ cells was incubated at 37°C for 2 hrs with 200 μM Ac-DEVD-pNA (caspase-3 substrate) or Ac-LEHD-pNA (caspase-9 substrate), and the absorbance of yellow pNA cleavage from its corresponding pre-cursors were measured using a spectrometer at 405 nm in a microplate reader (SpectraMax M2). The concentrations of total protein in supernatants were measured by Bradford method.

### Mitochondrial immunofluorescence staining

1 × 10^6^ cells/ml were plated onto the cover slips coated with 0.01% poly-l-lysine. After treatment, the cells were stained for 35 min. with 200 nM MitoTracker Red CMXRos (Molecular Probes, Carlsbad, CA, USA) and immunofluorescence staining was performed. Samples were visualized using a laser scanning confocal microscope (Leica).

### Preparation of cytosolic and mitochondrial fraction

Mitochondria were isolated as the standard protocol of the mitochondria extraction kits (Beyotime Biotechnology). Briefly, the cells were harvested and washed with ice-cold PBS and re-suspended in cytosol extraction buffer mix and incubated on ice for 10 min. The cells were homogenized in 1:10 isolation buffer and centrifuged for 10 min. at 700 × g and the supernatant was further centrifuged at 10,000 × g for 30 min. at 4°C. At this stage, the supernatant was collected and labelled as the cytosolic fraction. The pellet was suspended in mitochondrial extraction buffer, mixed by vortexing and labelled as the mitochondrial faction. Both cytosolic and mitochondrial factions were used to determine the release of cytochrome c (Cyt c), Bcl-2, Bcl-XL, Bax, Bad by western blotting as described below.

### Western blotting

Total protein samples containing 20 μg protein were subjected to 10% SDS-PAGE, transferred onto polyvinylidene difluoride membranes, and blocked with 7% non-fat milk in Tris-buffered saline and Tween 20 [TBST; 50 mM Tris-HCl (pH 7.6), 150 mM NaCl, 0.1% Tween-20] for 1.5 hrs at room temperature. Membranes were washed three times with TBST buffer and incubated at 4°C overnight with rabbit antibodies specific for rat Cyt c, Bcl-2, Bcl-XL, Bax, Bad, Nrf-2, P53, Puma. After washing with TBST, membranes were incubated with HRP-labelled goat antirabbit IgG (1:5000; Beijing Zhong Shan-Golden Bridge Technology Co., Ltd., Beijing, China) for 1.5 hrs at room temperature. Membranes were then washed with TBST, incubated with ECL reagent and exposed. Membranes were subsequently stripped and re-probed with GAPDH, COXIV or Actin antibody (1:500), which served as loading control.

### Statistical analysis

Data are expressed as the mean ± SEM of the indicated number of independent experiments. Student’s *t*-test and one-way anova were used to determine significance, with *P* < 0.05 considered significant. The Kaplan–Meier method was used for survival analysis with a log-rank of *P* < 0.05 to determine significance. Statistical analyses were performed with SPSS 10.0 (IBM SPSS Statistics company, Chicago, IL, USA) for Macintosh.

## Results

### Identification of the animal model and cell model

**Figure 1:**
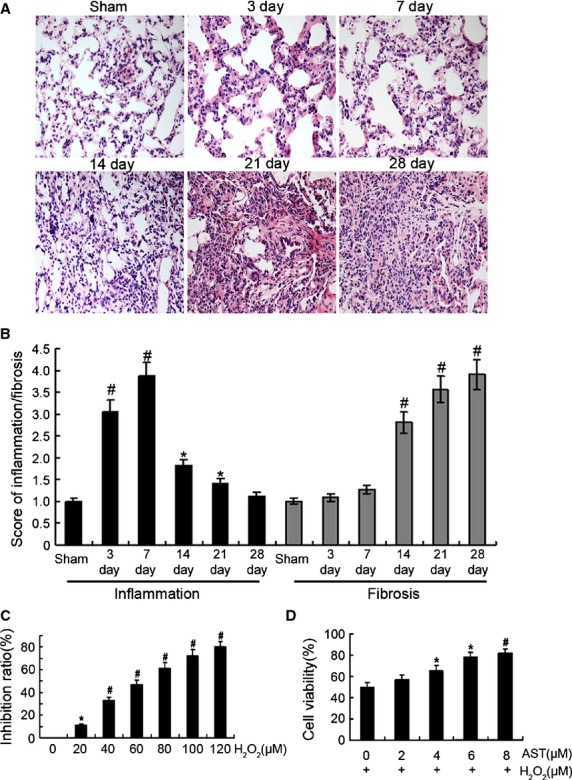
Identification of the animal model and cell model. (**A**) The pathogenesis of BLM-induced pulmonary fibrosis from 0 to 28 days after BLM administration by haematoxylin and eosin staining. Original magnification, ×400. (**B**) Grade of lung fibrosis and inflammation from 0 to 28 days after BLM administration. Histological grading of lesions was performed with the Szapiel method for extent of inflammation in lung parenchyma. Degrees of microscopic interstitial inflammation and fibrosis were graded on a scale of 1–4. (1), absent and appears normal (–); (2), light (+); (3), moderate (+ +) (4), strong (+ + + +). In addition, total lung inflammation and fibrosis score were calculated as the sum of the two components. Three sections collected from each lung were analysed in the experiment. Each bar represents the mean ± SD, *n* = 6. (**C**) H_2_O_2_ inhibition evaluated by SRB assay. RLE-6TN cells were treated with different concentrations of H_2_O_2_ for 12 hrs. (**D**) Effect of AST on cell viability as evaluated by SRB assay. Cells were pre-treated with different concentrations AST for 12 hrs and then co-treated with 65 μM H_2_O_2_ for 12 hrs. *P < 0.05; #*P* < 0.01.

Lung fibrosis is common associated with inflammation triggered by overproduction of free radicals in the early stage, and followed by increased expression of fibrotic markers. So, early stage is usually chosen to study the oxidative stress. We tested the pathogenesis of BLM-induced pulmonary fibrosis from 0 to 28 days using haematoxylin and eosin method (Fig. 1A and B). The data show that inflammation occurred in the early stage and fibrosis in the late stage. Therefore, 7 day model was selected for further studies.

Given that H_2_O_2_ has been reported as an oxidative agent 22, we tested whether H_2_O_2_ would affect RLE-6TN viability. We incubated RLE-6TN for 12 hrs under increasing concentrations of H_2_O_2_ and then evaluated the effect of H_2_O_2_ on RLE-6TN viability. With a resulting viability of 50 ± 0.21% for the cells exposed to 65 μM H_2_O_2_ for 12 hrs, these conditions were therefore selected for the rest of the experiments (Fig. 1C). To investigate the antioxidant capacity of AST on RLE-6TN, the cells were treated with different concentrations of AST for 12 hrs and then co-treated with 65 μM H_2_O_2_ for 12 hrs. Cell viability increased significantly to 81.2 ± 0.17% with 8 μM AST (Fig. 1D), so 8 μM AST was selected for further studies. These results indicate that the viability of H_2_O_2_-treated cells decreased significantly, and AST exerted a protective effect against H_2_O_2_-induced cytotoxicity.

### AST prevented apoptosis of AECs-II *in vivo and in vitro*

**Figure 2:**
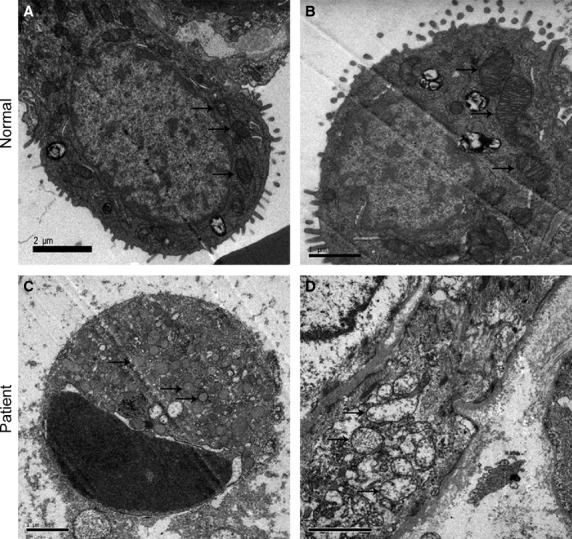
Apoptotic AECs-II and mitochondria observed in patient with lung fibrosis under TEM. Mitochondria were marked by arrows. (**A**) AECs-II observed in the normal under TEM. (**B**) Mitochondria observed in the normal under TEM. (**C**) Apoptotic AECs-II observed in patient under TEM. Crescent-shaped and condensed chromatin margined in the nuclear membrane. (**D**) TEM images show changes in the morphology of the mitochondria in patient, which had swollen and vacuole-like structures.

**Figure 3:**
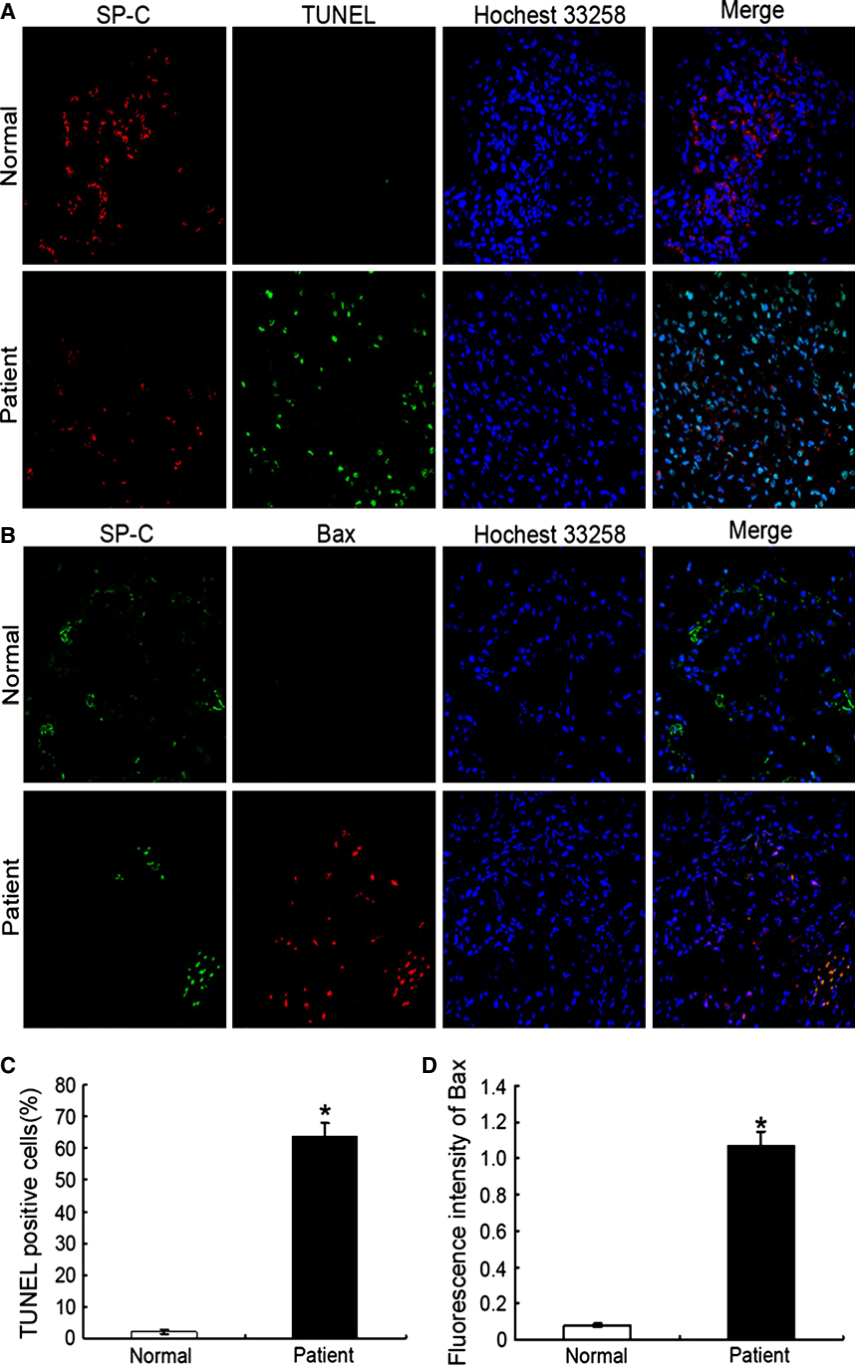
Apoptotic AECs-II observed in patient with lung fibrosis under a laser scanning confocal microscope. (**A**) Nuclear DNAs in the lung tissue of patient were observed using TUNEL. Condensation and fragmentation of nuclear DNA stained with FITC (green). The nuclei and SP-C were counterstained with Hoechst 33342 (blue) and SP-C antibodies (red), respectively. (**B**) Expression of bax in the lung tissue of patient. (**C**) TUNEL-positive cells increased in the lung tissue of patient. The TUNEL + SPC + cells and SPC+ cells in each whole image were counted in each whole image and bar graph represents the average value (TUNEL +SPC+ cells/SPC+ cells) from there independent experiments. (**D**) Expression of bax increased in the lung tissue of patient. The mean fluorescence intensity of Bax in each whole image was automatically quantified by Image-Pro Plus software and expressed in fluorescence units (FU) and bar graph represents the average value from there independent experiments; *P < 0.05.

The apoptosis of AECs-II is recognized as a critical event that initiates and propagates fibrosis in the lung parenchyma. The apoptotic AECs-II of lung tissues from patients were examined using TEM and a laser scanning confocal microscope. The results show that the apoptosis of AECs-II occurred in patients with lung fibrosis (Fig. 2). The apoptotic cell became small and round, the nuclear membrane had a crescent shape and condensed chromatin along its margin, the cytoplasm was compacted, and the organelle was compressed. TUNEL-positive cells showed condensed and fragmented nuclear DNAs in the lung tissues of patients (Fig. 3A and C). A few TUNEL-positive cells were detected in the control samples. To confirm these cells are AECs-II, we tested SP-C which is the AECs-II specific marker. The overlay results show that these DNAs were present in the cell nucleus of AECs-II. Expression of bax also increased in the AECs-II of patients (Fig. 3B and D).

**Figure 4:**
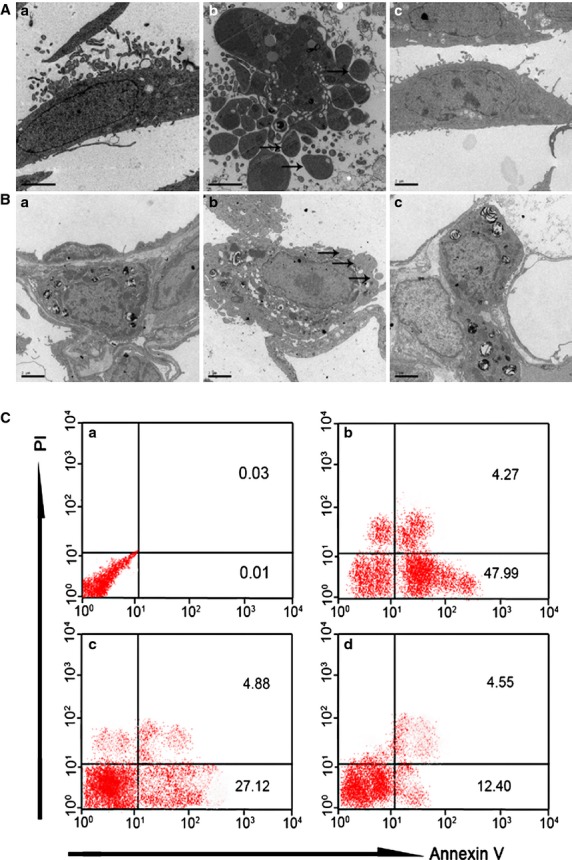
AST inhibited the apoptosis of AECs-II *in vivo and in vitro*. (**A**) TEM images of RLE-6TN cells: (**a**) Normal cells. (**b**) Apoptotic cells underwent apoptosis after 12 hrs H_2_O_2_ exposure alone. The apoptotic body was formed (marked by arrows). (**c**) Cells were treated with 8 μM AST alone for 12 hrs and co-treated with 65 μM H_2_O_2_ for 12 hrs. (**B**) TEM images of AECs-II in the animal model: (**a**) AECs-II in the sham group. (**b**) Apoptotic AECs-II in BLM group was characterized by blebbing of the cell membrane (marked by arrows). (**c**) AECs-II in AST II group. (**C**) AST reduced the apoptosis ratio in RLE-6TN cells. The cells were labelled with Annexin V-FITC antibody and propidium iodide (PI) and then analysed using flow cytometry. Early apoptotic cells with intact cell membranes bound to Annexin V-FITC but excluded PI. The cells in late apoptotic or necrotic stages were labelled using both Annexin V-FITC and PI. The pixels at the bottom-right quadrant of each panel show V-FITC-positive and PI-negative cells, indicating that the cells were in an early stage of apoptosis. The pixels at the top-right quadrant represent the cells stained with both PI and Annexin V, indicating that the cells were in late apoptosis or were no longer viable. (**a**) Normal RLE-6TN cells. (**b**) Cells were treated with 65 μM H_2_O_2_ for 12 hrs. (**c**) Cells were co-treated with 65 μM H_2_O_2_ and 8 μM AST for 12 hrs. (**D**) Cells were treated with 8 μM AST alone for 12 hrs and then co-treated with 65 μM H_2_O_2_ for 12 hrs.

The apoptosis of AECs-II in actual patients was similar to that in the cell and animal model, indicating that our oxidative stress model was significant *in vivo and in vitro* (Fig. 4). The TEM results show that the cell and nuclear membranes were clear-cut and integrated in normal AECs-II. In H_2_O_2_ or bleomycin (BLM)-treated AECs-II, the cell membrane was incomplete and apoptotic body was formed, which was improved by AST. Flow cytometry was used to determine the apoptosis ratio in RLE-6TN cells under different conditions. The data indicated that 47.99% of the cells underwent early apoptosis after 12 hrs H_2_O_2_ alone exposure, which was reduced to 27.12% when the cells were co-treated with AST and H_2_O_2_ for 12 hrs, and 12.40% when the cells were treated with AST alone for 12 hrs and then co-treated with H_2_O_2_ for 12 hrs. These findings show that AST could prevent apoptosis of AECs-II *in vivo and in vitro*.

### AST contributed to the protection against oxidative stress *in vivo and in vitro*

**Figure 5:**
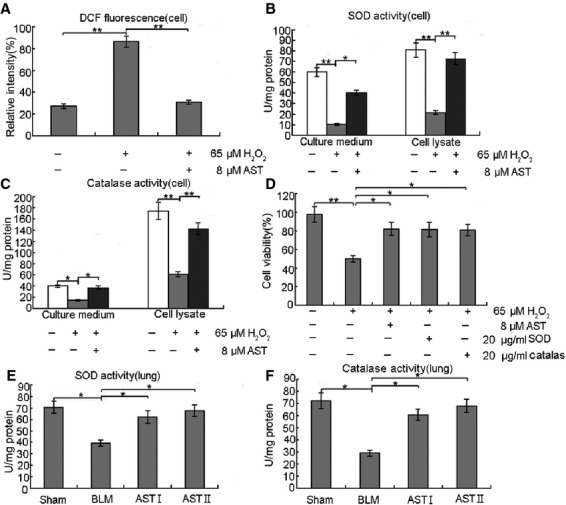
Antioxidative properties of AST facilitated protection from oxidative stress *in vitro and in vivo*. (**A**) RLE-6TN cells were treated with 65 μM H_2_O_2_ alone for 12 hrs and co-treated with 8 μM AST for 24 hrs. After treatment, ROS generation was determined using 5 μM CMH2DCFDA. AST exhibited significantly reduced H_2_O_2_-mediated ROS generation in RLE-6TN cells. (**B** and **C**) RLE-6TN cells were treated with 65 μM H_2_O_2_ alone for 12 hrs and co-treated with 8 μM AST for 24 hrs. SOD and catalase activities were measured after treatments. AST significantly increased SOD and catalase activities. (**D**) RLE-6TN cells were treated with 65 μM H_2_O_2_ alone for 12 hrs and co-treated with 8 μM AST, 20 μg/ml SOD or 20 μg/ml catalase for 24 hrs. AST, SOD and catalase improved cell viability. (**E** and **F**) The lung tissues were homogenated, centrifuged at 5000 × g for 15 min. at 4°C, and the supernatant was got to detect SOD and catalase. AST also increased SOD and catalase activities *in vivo*. *P < 0.05 and **P < 0.01, compared with the group treated with H_2_O_2_ or BLM alone.

To determine the suppression of H_2_O_2_-induced ROS production using AST, we measured the ROS production in RLE-6TN cells under several conditions. ROS generation, which was detected in the H_2_O_2_-treated cells, was suppressed by AST treatment (Fig. 5A). These results suggest that AST acts as an inhibitor (antioxidant) to H_2_O_2_-mediated ROS generation. We hypothesized that H_2_O_2_ generates ROS by inhibiting antioxidant enzymes, such as SOD and catalase, and AST suppresses H_2_O_2_-induced ROS generation by protecting these antioxidant enzymes from H_2_O_2_. To examine this possibility, cells were treated with H_2_O_2_ in the presence or absence of AST. Then, the SOD (Fig. 5B) and catalase activities (Fig. 5C) were determined. The decrease in SOD and catalase activities after H_2_O_2_ treatment are shown in Figure 4B and C, respectively. The inhibitory effect of H_2_O_2_ on SOD and catalase activities suggests that the cytotoxic effect of H_2_O_2_ may have resulted from the oxidative stress in RLE-6TN cells. This possibility was examined by adding SOD or catalase protein during H_2_O_2_ treatment. Cell viability decreased to 50% when the cells were treated with H_2_O_2_ alone for 12 hrs, but this H_2_O_2_-induced viability loss was almost fully inhibited by AST, SOD and catalase (Fig. 5D). The antioxidative properties of AST *in vivo* showed similar effects as those shown in Figure 5E and F. Based on these findings, we hypothesized that the antioxidative properties of AST may contribute to the protection from oxidative stress.

### AST improved the condition of the AECs-II mitochondria *in vivo and in vitro*

**Figure 6:**
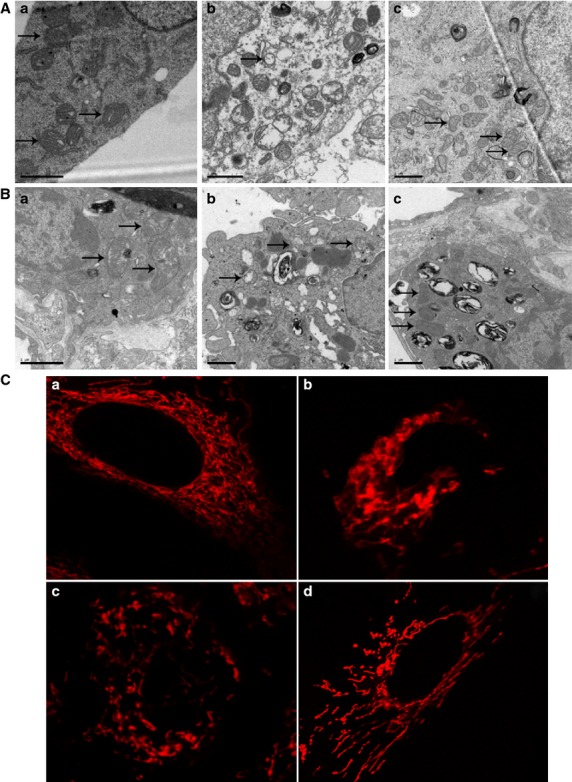
AST improved the mitochondria of AECs-II *in vivo and in vitro*. Mitochondria were marked by arrows. (**A**) TEM images of mitochondria in RLE-6TN cells. (**a**) Mitochondria in normal RLE-6TN cells. (**b**) Abnormal mitochondria in RLE-6TN cells after exposure to H_2_O_2_ for 12 hrs. (**c**) Improved mitochondria in RLE-6TN cells treated with 8 μM AST alone for 12 hrs and co-treated with 65 μM H_2_O_2_ for 12 hrs. (**B**) TEM images of AECs-II mitochondria in the animal model. AECs-II mitochondria in the (**a**) sham, (**b**) BLM and (**c**) AST groups. (**C**) Mitochondrial arrangement in RLE-6TN cells under a laser scanning confocal microscope. (**a**) Mitochondria arranged orderly in normal cells. (**b**) Mitochondria arranged disorderly after 65 μM H_2_O_2_ treatment for 12 hrs. (**c** and **d**) Mitochondrial arrangement improved after co-treatment with 65 μM H_2_O_2_ for 12 hrs and 8 μM AST for 12 and 24 hrs.

**Figure 7:**
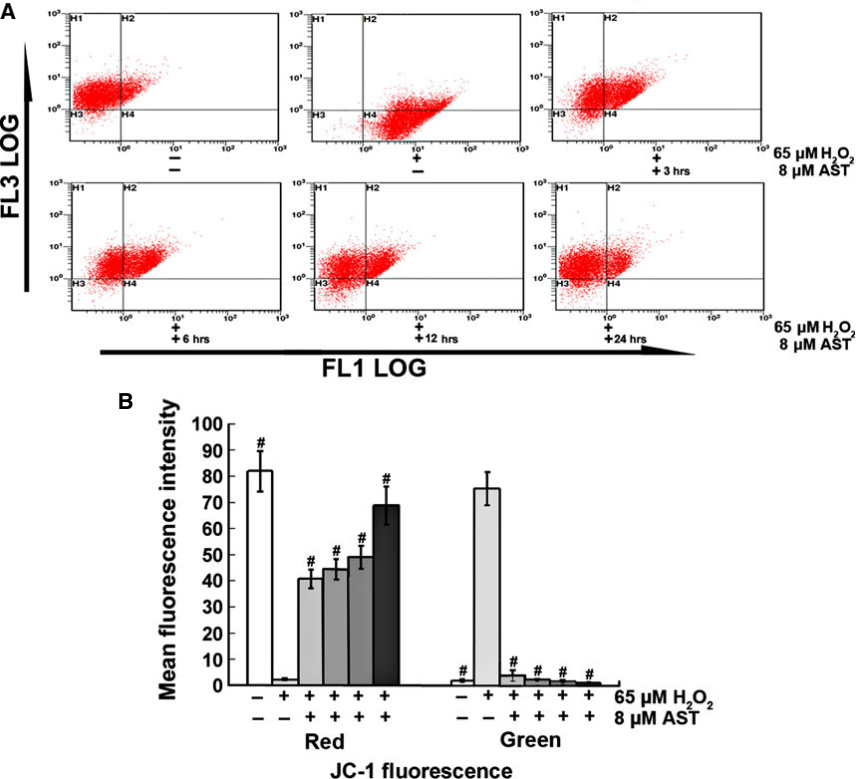
AST increased MMP of RLE-6TN cells. (**A**) Representative dot plot of the changed MMP using flow cytometry after labelling the fluorescent probe with JC-1. FL1-LOG Green, FL3-LOG Red; Green fluorescence represents the monomeric form of JC-1 with low MMP. Red fluorescence represents the mitochondrial aggregate form of JC-1 with high MMP. (**B**) Percentages of red and green fluorescence intensities from different groups. The data are expressed as mean ± SD from six independent experiments, #*P* < 0.01 *versus* control cells treated with H_2_O_2_ alone.

To further illustrate the improvement of the mitochondria of AECs-II by AST *in vivo and in vitro*, we observed the changes in mitochondrial morphology using TEM (Fig. 6A and B), mitochondrial arrangement using immunofluorescence (Fig. 6C), and then measured MMP using flow cytometry (Fig. 7). TEM results show that the structural integrity of the mitochondrial inner and outer membranes and the mitochondria cristae were not affected in the normal groups *in vivo and in vitro*. The mitochondria of H_2_O_2_- or BLM-treated AECs-II showed an altered membrane structural integrity. Some of the mitochondria were swollen with a vacuole-like structure, some even showed a lower amount number of cristae or deformed cristae, and few mitochondria had cristae parallel to the long axis. However, AST improved these changes in the morphology of the mitochondria. Mitochondrial immunofluorescence staining results show that the orderly arrangement of the mitochondria was disrupted after H_2_O_2_-treatment. However, AST improved this disordered state. The MMP results from Figure 6 show that H_2_O_2_ reduced MMP in RLE-6TN cells, as indicated by a decrease in red and increase in green cells. However, AST increased MMP in a time-dependent manner.

### AST prevented the translocation of apoptotic proteins

**Figure 8:**
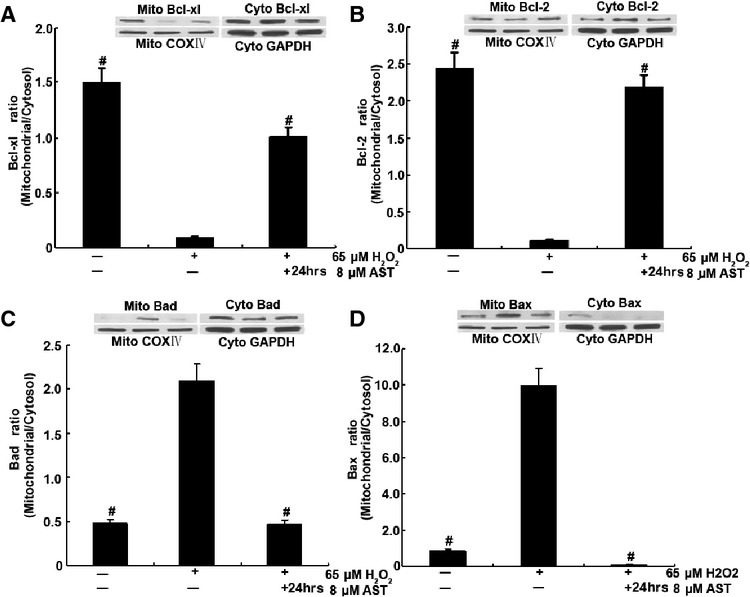
AST prevented H_2_O_2_-induced translocation of Bcl-2 family proteins in RLE-6TN cells. Cells were treated with 8 μM AST alone for 12 hrs and then co-treated with 65 μM H_2_O_2_ for 12 hrs. Then, cytosolic and mitochondrial fractions were prepared, and Bcl-2 member proteins were detected using specific antibodies by immunoblot. A representative blot from duplicate experiments is shown. #*P* < 0.01 *versus* cells treated with H_2_O_2_ alone.

Cellular apoptosis is mediated by a fine balance of pro- and antiapoptotic proteins in cells. Specifically, the mitochondrial apoptosis pathway is regulated by early translocation of antiapoptotic (Bcl-2 and Bcl-xl) and pro-apoptotic (Bad and Bax) members of the Bcl-2 family of proteins to or from the mitochondria. We therefore investigated in RLE-6TN the translocation of Bcl-2 member proteins to the mitochondria or the cytosol after incubation with H_2_O_2_ in the absence or presence of AST (Fig. 8). As shown in Figure 8, H_2_O_2_ caused the translocation of pro-apoptotic members such as Bad and Bax to the mitochondria and that of antiapoptotic members to the cytosol. These changes were significantly prevented in H_2_O_2_ and AST-co-treated RLE-6TN cells. These results indicate that H_2_O_2_ activates the mitochondrial apoptotic pathway, but AST prevents it.

### AST inhibited Cyt c release and caspase-9 and -3 activation

**Figure 9:**
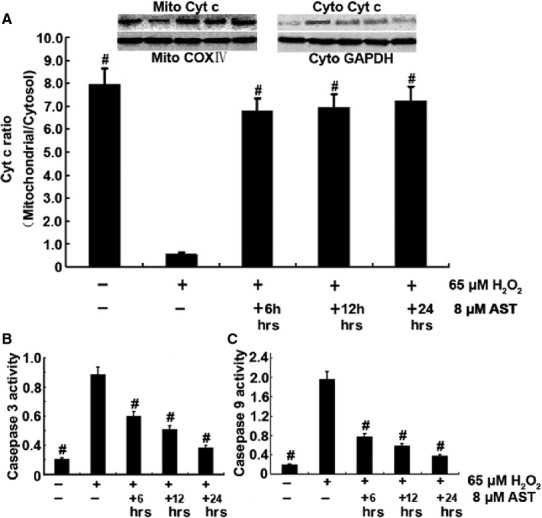
AST suppressed Cyt c release and caspase-3 and -9 activation. 6 hrs AST group: Cells were treated with 65 μM H_2_O_2_ alone for 6 hrs and co-treated with 8 μM AST for 6 hrs. 12 hrs AST group: Cells were co-treated with 65 μM H_2_O_2_ and 8 μM AST for 12 hrs. 24 hrs AST group: Cells were treated with 8 μM AST alone for 12 hrs and then co-treated with 65 μM H_2_O_2_ for 12 hrs. (A) H_2_O_2_ altered the mitochondrial functions, as indicated by the increased release of Cyt c from the mitochondria to the cytosol. AST prevented Cyt c release to the cytoplasm. AST inhibited caspase-3 (B) and -9 activation (C). #*P* < 0.01 *versus* cells treated with H_2_O_2_ alone.

The translocation of apoptotic proteins regulates the release of pro-apoptotic molecules, such as Cyt c, that activate the effecter caspases. The release of Cyt c from the mitochondria into the cytosol is a major apoptosis pathway. Cytosolic Cyt c induces the caspase-9-dependent activation of caspase-3. To elucidate whether Cyt c release occurs in H_2_O_2_-induced apoptosis, we determined the activities of Cyt c using western blot (Fig. 9A). The immunoblot results showed that the cytosolic Cyt c levels increased after 65 μM H_2_O_2_ treatment, and AST gradually decreased the cytosolic Cyt c levels in a time-dependent manner. The Cyt c levels markedly increased in the cytosolic fraction and decreased in the mitochondrial fraction in the H_2_O_2_ treatment alone group. By contrast, the Cyt c levels markedly decreased in the cytosolic fraction and increased in the mitochondrial fraction after the 24 hrs AST treatment. AST administration increased the level of Cyt c in the mitochondrial fraction and reduced that in the cytosol, thereby significantly preventing Cyt c release. Furthermore, we examined the activities of caspase-9 and -3 using a microplate reader (Fig. 9B and C). Contrary to the H_2_O_2_-treated cells, the AST-treated cells had decreased activities of caspase-9 and -3.

### AST activated Nrf-2 and other cytoprotective genes

**Figure 10:**
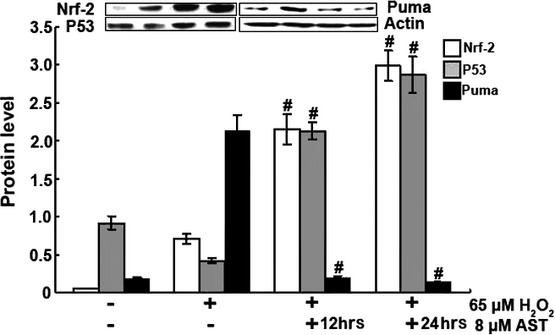
AST activated Nrf-2 and P53 and suppressed Puma, as indicated by Western blot. 12 hrs AST group: Cells were co-treated with 65 μM H_2_O_2_ and 8 μM AST for 12 hrs. 24 hrs AST group: Cells were treated with 8 μM AST alone for 12 hrs and then co-treated with 65 μM H_2_O_2_ for 12 hrs. #*P* < 0.01 *versus* control cells treated by H_2_O_2_ alone.

Nrf-2 is a key transcriptional regulator for the protection of cells against oxidative stresses. The profound oxidative effect by H_2_O_2_ and its inhibition by AST in RLE-6TN cells raised the possibility that Nrf-2 is involved in the actions of H_2_O_2_ and AST. As shown in Figure 10, AST strongly and H_2_O_2_ weakly increased the protein level of Nrf-2 in RLE-6TN cells, indicating Nrf-2 activation. Co-treatment with H_2_O_2_ and AST further increased Nrf-2 protein, suggesting a co-operation between H_2_O_2_ and AST for Nrf-2 activation. Western blot was performed to analyse induction of P53 and Puma, two genes related with mitochondria and Nrf-2. The data indicate that AST affected the protein levels of P53 and Puma *in vitro*. P53 gradually increased in the AST-treated groups, whereas Puma gradually decreased under the same conditions. Therefore, AST activated the mitochondrion-mediated signal transduction to induce cytoprotective genes.

## Discussion

Apoptosis plays different roles because different types of cells exist in lung fibrosis 23. The first role may be beneficial to the body by inducing the apoptosis of inflammatory cells to halt the inflammation of and damage to lung tissue. The second function could be deleterious by inducing the apoptosis of AECs and destroying the alveolar structure. Apoptosis of AECs-II is increasingly being recognized as a critical event that initiates and propagates fibrosis in lung parenchyma 23–25. The concept that AECs-II death is a critical determinant of fibrosis *versus* normal repair has long been proposed on the basis of two-hit toxicological experiments that demonstrated that fibrogenesis could be induced by experimental delay of epithelial repair after lung injury 26,27, regardless of the presence or absence of inflammation. More recent evidence in support of this theory was found in the ability of caspase inhibitors 28,29 or the genetic deletion of apoptosis signalling molecules 30 to block fibrogenesis subsequent to lung injuries aimed at the epithelium 31. Understanding the regulation of AEC-II apoptosis is therefore critical to understanding the pathogenesis of lung fibrosis. In the present study, we observed the apoptosis of AECs-II in patients with lung fibrosis, which were consistent with the results from previous studies 32,33. Different models of pulmonary fibrosis have been developed over the years. Most of them mimic some, but never all features of human pulmonary fibrosis, especially the progressive and irreversible nature of the condition. In apoptosis aspect our animal and cell models are similar to patients’ condition, so the study is meaningful for clinical testing of new drugs. AST as an antioxidant, is widely used in food and nutrition, but no data supporting their validity as an experimental model of human pulmonary fibrosis. Our results show that AST provides protection from oxidative stress-induced apoptosis through the mitochondrial signalling pathway. No combined study of all these systematic apoptosis-regulatory molecules by AST has been performed. Our findings support the notion that apoptosis could be a key element in the pathogenesis of lung fibrosis and indicate the possible pathways implicated in this disease as future targets of therapeutic attempts.

So far, BLM is the standard agent for induction of experimental lung fibrosis in animals. Over the years, numerous agents have been tested to anti-inflammatory, antioxidant and anti-fibrotic effects in this model 34. The initial elevation of inflammation is followed by increased expression of fibrotic markers in this model. It causes inflammatory and fibrotic reactions within a short period of time. The ‘switch’ between inflammation and fibrosis appears to occur around day 9 after BLM 35. BLM causes an inflammatory response which triggered by overproduction of free radicals, so we choose 7 day BLM lung fibrosis model to investigate the antioxidative effect of AST and 28 day model to investigate anti-fibrotic effect 17.

Oxidative stress is a common phenomenon in the pathological processes of various respiratory diseases, including lung fibrosis, which is a severe, clinical, nonreversible and pathological lung injury without effective therapy. Lung fibrosis exhibits various pathological features, including an accumulation of inflammatory cells, epithelial injury and collagen deposition. However, ROS bursting from inflammations and the disruption of the body’s antioxidant defence systems have been suggested as possible causes of early injuries in lung fibrosis 36, which in turn contributes to DNA chain breakage and lipid peroxidation of the cell membrane 37. SOD and catalase are important antioxidants in cellular redox disequilibrium in fibrogenesis, in which they coordinate on the detoxification of superoxide and H_2_O_2_/BLM to form water and molecular oxygen. Typically, the large amount of ROS induced by xenobiotics exhausts and decreases SOD and catalase activities in cells or tissues. As expected, a notable decline in the activity of these enzymes and an increase in ROS generation were evident in the H_2_O_2_- and BLM-induced lung fibrosis models used in this study. AST protects against H_2_O_2_- or BLM-induced oxidative injury by reducing ROS generation and increasing SOD as well as catalase activity.

To investigate the effects of AST inhibition of the early apoptotic events, we examined the translocation of the Bcl-2 family proteins in cells. The mitochondrial apoptosis pathway is known to be regulated by members of the Bcl-2 family of proteins, which includes both antiapoptotic (Bcl-2 and Bcl-xl) and pro-apoptotic (Bad and Bax) members 38. When stimulated by oxidants, such as H_2_O_2_, Bad translocates to the mitochondria and removes the inhibitory effects of Bcl-2 on Bax, which in turn leads to the formation of Bax–Bax dimer pores on the outer membrane. These pores can alter mitochondrial permeability, which in turn results in the release of pro-apoptotic molecules such as Cyt c. Changes in mitochondrial permeability lead to the release of Cyt c from the mitochondria, which is required for the hallmarks of cytosolic and nuclear apoptosis, such as caspase-3 and -9 activation 39. Our results show that AST prevents the translocation of the pro-apoptotic proteins Bax and Bad to the mitochondria and that of Bcl-2 from the mitochondria to the cytosol. We have also demonstrated in this study that the release of Cyt c into the cytosol, which may lead to eventual apoptosis 40, is inhibited by AST. The effect of AST inhibition of apoptosis *in vivo and in vitro* through the mitochondrial signalling pathway was further shown in this study by mitochondrial immunofluorescence staining, mitochondrial observation under TEM, MMP assay and apoptosis ratio.

To maintain a proper redox balance, the respiratory system is endowed with an antioxidant defence system consisting of endogenous antioxidant enzymes. The expression of most antioxidant enzymes is tightly controlled by the antioxidant response element and is activated by Nrf-2, a redox-sensitive transcription factor 41. Previous studies have highlighted the protective effects of Nrf-2 activation in reducing oxidative stress in many pulmonary disorders 42,43. In the lungs, the dysfunction of cellular defence systems resulting from Nrf-2 removal has been proven to increase animals’ susceptibility to acute lung injury 44 or pulmonary emphysema 45. An important series of papers from Kleeberger’s Laboratory have reported that Nrf-2 deficiency in the lungs exacerbates the toxicity caused by multiple oxidative insults, including hyperoxia 46, viruses 47, or BLM 48. Thus, the critical role of Nrf-2 in regulating pulmonary disease is clearly demonstrated. However, direct evidence of the role of Nrf-2 in fibrotic processes and the pathway through which it is regulated during AST treatment remains to be investigated. Our data showed that AST increases Nrf-2 level suggests that AST ameliorates oxidative stress by activating Nrf-2.

Nrf-2 facilitates the induction of target genes responsible for cell survival (*i.e*., Bcl-2 family) 49–51. When cells are exposed to H_2_O_2_, Nrf-2 is activated as an adaptive response to escape from oxidative stress 52. However, Nrf-2 activated by H_2_O_2_ limits the capacity of cells to escape from injury, because they also stimulate cell death signalling. In our results, the expressions of caspase-3 and -9, P53, Puma, and the Bcl-2 family illustrate this behaviour. Thus, we examined the expression of P53 and Puma, the two most thoroughly studied factors involved in the mitochondrial signalling pathway. This concept was strengthened by the ability of AST to improve p53 and inhibit Puma. Interestingly, expression of P53 was increased after AST treatment. Links between ROS and p53 activity have previously been reported. Namely, activated p53 increases cellular ROS by enhancing the transcription of pro-apoptotic genes. Moreover, scavenging of ROS by antioxidant therapy decreases apoptosis induced by p53. Paradoxically, other studies provide evidence of an antioxidant role of p53 that protects against oxidative DNA damage and genomic instability. In this way our result seems to support that the relative pro-apoptotic and antiapoptotic functions of p53 would appear to depend on the cellular p53 concentration, subcellular localization, phosphorylation status or protein partner interactions 53,54.

**Figure 11:**
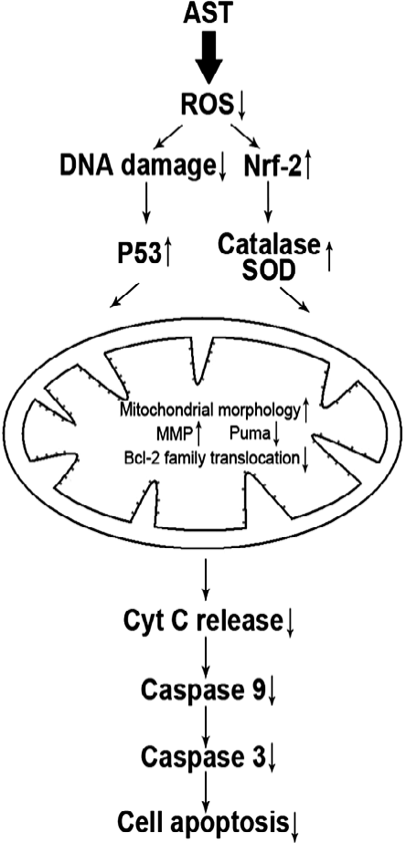
Proposed pathway of the antioxidative effects of AST through the mitochondrion-mediated signalling pathway of apoptosis. H_2_O_2_ or BLM induces ROS formation, which results in DNA damage and leads to apoptosis. AST decreases ROS formation and blocks H_2_O_2_- or BLM-induced apoptosis by inhibiting the translocation of Bad and Bax to the mitochondria and that of Bcl-2 and Bcl-XL to the cytosol. This phenomenon decreases the expression of Puma and increases the expression of MMP, subsequently inhibiting the release of pro-apoptosis molecules causing caspase activation, which leads to apoptosis.

Although the precise pathway of how AST inhibits H_2_O_2_- or BLM-mediated apoptosis is not known, we hypothesize that the inhibition of ROS by AST could be involved in the activation of a redox-sensitive signalling cascade. AST may prevent apoptosis by blocking the activation of mitochondrion-mediated signalling intermediates in addition to maintaining proper redox balance, as demonstrated by the present findings (Fig. 11). Hence, our results provide evidence that AST prevents H_2_O_2_-induced subcellular translocation of apoptogenic Bcl-2 family proteins and modulates cytotoxic signals by inhibiting ROS formation and the subsequent activation of cytoprotective signals in AECs-II. These data provide a rationale for exploring the therapeutic and chemoprotective potentials of AST for the treatment and prevention of pathologic conditions involving pulmonary fibrosis in future studies.
